# Langmuir–Blodgett Films of Conjugated Polymer and Silver Nanoparticles: A Possible Substrate for Pesticide SERS-Based Detection

**DOI:** 10.1021/acs.langmuir.5c03648

**Published:** 2025-09-09

**Authors:** Rebeca da Rocha Rodrigues, Diogo Silva Pellosi, Luciano Caseli, Laura Oliveira Péres

**Affiliations:** † 28105 Federal University of São Paulo, Laboratory of Hybrid Materials, Diadema, São Paulo 09913-030, Brazil; ‡ 28122 Federal University of Paraná, Macromolecules and Interfaces Research Group, Curitiba, Paraná 81531-980, Brazil

## Abstract

This study demonstrates the successful fabrication of nanostructured Langmuir–Blodgett (LB) films combining the conjugated copolymer poly­(9,9-dioctylfluorene-*co*-3,4-ethylenedioxythiophene) (PDOF-*co*-PEDOT) with spherical and triangular silver nanoparticles (AgNP). The LB technique allowed precise control over the molecular arrangement and distribution of the nanoparticles at the air–water interface, resulting in compact, reproducible and structurally ordered nanocomposite films. The structural and morphological properties of the interfacial monolayers and LB films were investigated using surface pressure–area isotherms, Brewster angle microscopy, polarization modulation infrared reflection–absorption spectroscopy (PM-IRRAS) and quartz crystal microbalance. Monolayers and LB films containing anisotropic nanoparticles exhibited greater restriction in the molecular reorganization of the monolayers and in the degree of transfer and organization of the LB films. Physicochemical characterization confirmed that the organization of the films at the molecular level plays a key role in improving the performance of surface-enhanced Raman scattering (SERS). Films with spherical AgNP showed relatively intense and well-defined Raman signals for the pesticide chlorpyrifos (CLP), even at concentrations less than 6 × 10^–6^ mol L^–1^, outperforming analogous films prepared by spin coating. In contrast, films with triangular AgNP showed lower spectral reproducibility due to their lower colloidal and structural stability. The results show that the LB technique enables effective nanostructuring and synergistic integration of conducting polymers and plasmonic nanoparticles and offers a promising route to the development of SERS-active platforms. To our knowledge, this is one of the first studies to investigate the use of conjugated polymers as functional matrices and silver nanoparticles organized by the LB technique that can be exploited for SERS-based detection. This work also contributes to the under-explored field of hybrid LB films for chemical detection by highlighting their potential for the construction of highly structured and functionalized interfaces for molecular recognition.

## Graphical abstract

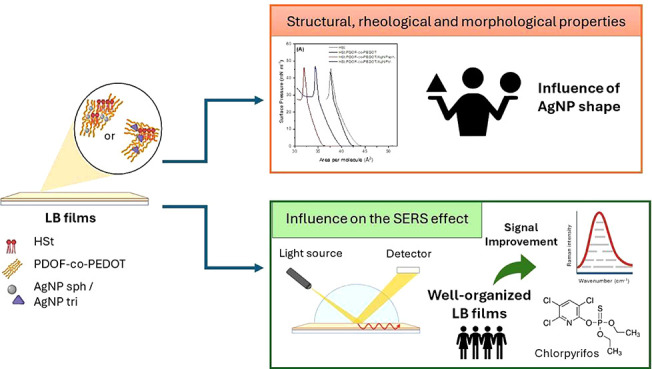

## Introduction

Polymers are materials that are used in many everyday applications. Among them, conjugated polymers (CPs) stand out for technological applications due to their ability to conduct electricity. This property results from the presence of alternating single and double bonds along the backbone, allowing π-electrons to be delocalized along the polymer chain.^1,2^ Among CPs, polythiophenes and their derivatives have attracted particular attention due to their thermal stability, ease of structural modification and good processability.^3,4^ One of the most promising examples is poly­(3,4-ethylenedioxythiophene) (PEDOT), which combines high electrical conductivity with excellent electrochemical stability.^5^ The compatibility of PEDOT and its strong adhesion to other materials make it a suitable matrix for the incorporation of various compounds, including metallic nanoparticles, which has led to its increasing use in the development of sensors.^6,7^ Another notable conjugated polymer is polyfluorene (PF), which exhibits strong fluorescence due to its planar biphenyl structure and π-electron-rich system. PF also offers excellent solubility, film formation, thermal and chemical stability, which makes them attractive for optoelectronic devices.^7,8^ In advanced polymer science, copolymers, polymers composed of two or more different monomers, stand out for their unique properties resulting from the synergy between their constituent monomers.^9^ Therefore, the copolymer composed of PEDOT and PF studied in this work could be a promising compound for application in devices and sensors.

A crucial aspect in the fabrication of CPs-based devices is the ability to precisely control the architecture and molecular organization of thin polymer films.^10,11^ Since the performance of such devices, including their sensitivity and selectivity as sensors, can be significantly improved by molecular ordering, techniques that promote high structural organization are of particular interest. In this context, the Langmuir–Blodgett (LB) technique stands out as a bottom-up approach that enables the formation of highly organized monolayers.^10–12^ In this method, an organic solution of an insoluble and amphiphilic substance is distributed over an air–water interface. After evaporation of the solvent, a monomolecular film is formed, the so-called Langmuir monolayer, which can be laterally compressed by movable barriers, thereby reducing the available surface area and promoting molecular packing. This organized monolayer can be transferred to solid substrates, resulting in films whose thickness is controlled by the number of times the substrate passes through the interface, allowing for architecture and tunable multilayer configurations.^10,11^

Originally, the LB technique was used for classic amphiphilic molecules such as lipids and fatty acids. It has since been extended to new materials such as metallic nanoparticles^12,13^ and CPs.^10^ When some of these components are combined in a single film, nanocomposites are formed and can exhibit synergistic properties that are particularly beneficial in sensing applications. Recent studies have shown that such hybrid films composed of plasmonic metallic nanoparticles (such as silver and gold) and CPs can significantly enhance Raman scattering signals, making them promising sensors for surface-enhanced Raman scattering (SERS) capable of detecting trace amounts of environmental pollutants such as pesticides.^14,15^ The dispersion of metal nanoparticles in the polymer matrix reduces nanoparticle aggregation/agglomeration processes and provides synergistic properties, resulting in SERS substrates with improved thermal stability, mechanical properties and increased responsiveness.^14,15^ Additionally, CPs offer precise control over the organization of metallic nanoparticles, promoting reproducible “hotspots”—regions where occurs extreme amplification of the electromagnetic field and consequently the Raman signal^16,17^ —and enhancing the SERS effect via the electromagnetic (EM) mechanism,^14^ acting not only as a mechanical support to the other sensor components, as frequently reported. Similar to semiconductor-based SERS substrates, CPs can also support charge transfer processes associated with the chemical (CM) mechanism, due to the presence of HOMO and LUMO orbitals.^14,18^ This coexistence of EM and CM mechanisms can further amplify the SERS response.^14,15^ Despite their potential, hybrid SERS substrates containing conjugated polymers remain scarcely explored, representing a promising path for future research.^14,19^ In this context, the use of CPs and the high degree of control over the molecular organization of monolayers and LB films can be highly beneficial for SERS substrates, as this technique allows better packing, arrangement, and interaction between the nanoparticles.^12,20,21^

Based on these findings, the present work investigates the interactions between the copolymer poly­(9,9-dioctylfluorene-*co*-3,4-ethylenedioxythiophene) (PDOF-*co*-PEDOT) and silver nanoparticles (AgNP) of two different morphologies (spheres and triangular plates) at the air–water interface. The study focuses on how these interactions affect the molecular organization of Langmuir and LB films and evaluates the potential of the resulting nanocomposite films for SERS-based detection of the pesticide chlorpyrifos (CLP). The focus is on understanding how the structural organization of the films affects their performance as SERS-active platforms for the detection of contaminants in aqueous media.

## Experimental Section

### Synthesis of Copolymer (PDOF-*co*-PEDOT) and Silver Nanoparticles (AgNP)

The copolymer poly­(9,9-dioctylfluorene-*co*-3,4-ethylenedioxythiophene) (PDOF-*co*-PEDOT) was synthesized according our earlier study,^22^ using the Suzuki route.^23^ The structural, optical and thermal properties of the obtained brown solid were analyzed by Fourier transform infrared spectroscopy (FTIR), UV–vis spectroscopy (UV–vis), fluorescence emission, X-ray photon electron spectroscopy (XPS), thermogravimetric analysis (TGA) and differential scanning calorimetry (DSC). Molar mass: Mw = 3.68 × 10^3^ g mol^–1^ and *M*_w_/*M*_n_ = 2.46.

The spherical and triangular silver nanoparticles (AgNP) were obtained by reduction of silver nitrate with sodium borohydride and sodium citrate as reducing and stabilizing agents, respectively.^22,24^ The glassware used for the synthesis was previously cleaned with a sulfuric acid/hydrogen peroxide solution (3:1 v/v) and then rinsed with ultrapure water. The AgNP dispersions were characterized by UV–vis, dynamic light scattering (DLS), zeta potential and transmission electron microscopy (TEM). The hydrodynamic diameter values obtained by DLS of spherical and triangular AgNP were 13.37 and 28.65 nm, respectively.^22^

As mentioned previously, the specific properties of the PDOF-*co*-PEDOT and AgNP were analyzed and the technical specifications were described in our previously published study.^22^ Gel permeation chromatography (GPC) of PDOF-*co*-PEDOT were obtained by Agilent 1100 chromatograph, using tetrahydrofuran as solvent and polystyrene solutions as standard.

### Preparation of Langmuir Films

Water purified with the Milli-Q/Millipore system (resistivity of 18.2 MΩ cm and surface tension of 72.8 mN m^–1^ at 20 °C) was used as the subphase for all Langmuir monolayers. Stearic acid (HSt) was purchased from Sigma-Aldrich and dissolved in chloroform (CHCl_3_) at the concentration of 0.5 mg mL^–1^. For the studies with PDOF-*co*-PEDOT floating films, aliquots of a 0.5 mg mL^–1^ solution of PDOF-*co*-PEDOT (in CHCl_3_) were carefully spread drop by drop on the air–water interface. After evaporation of the solvent (20 min), compression was performed with mobile barriers at a rate of 10 mm min^–1^ (7.5 cm^2^ min^–1^).

For the monolayers of PDOF-*co*-PEDOT with stearic acid (HSt), 70 μL solutions with different proportions of the copolymer and HSt (0.5 mg mL^–1^ in CHCl_3_) were spread on the air–water interface (Figure 1). For the monolayers with AgNP, aliquots of the AgNP dispersion were spread on the interface after application of the HSt:PDOF-*co*-PEDOT solution. An interval of 30 min was set for evaporation of the solvent and the stabilization of entire system of the mixed monolayers. Compression was carried out at the same rate as for the pure polymer floating films. All monolayers were characterized by surface pressure–area (π-*A*), surface potential-area (ΔV-A) isotherms and compression/decompression π-*A* cycles for hysteresis analysis. The surface pressure was measured using a Wilhelmy plate (filter paper – 10 × 20 mm). ΔV-A isotherms were co-obtained with π-*A* isotherms using a KSV NIMA Surface Potential Sensor (SPOT), based on Kelvin vibrating probe method, using a platinum counter electrode.

**Figure 1:**
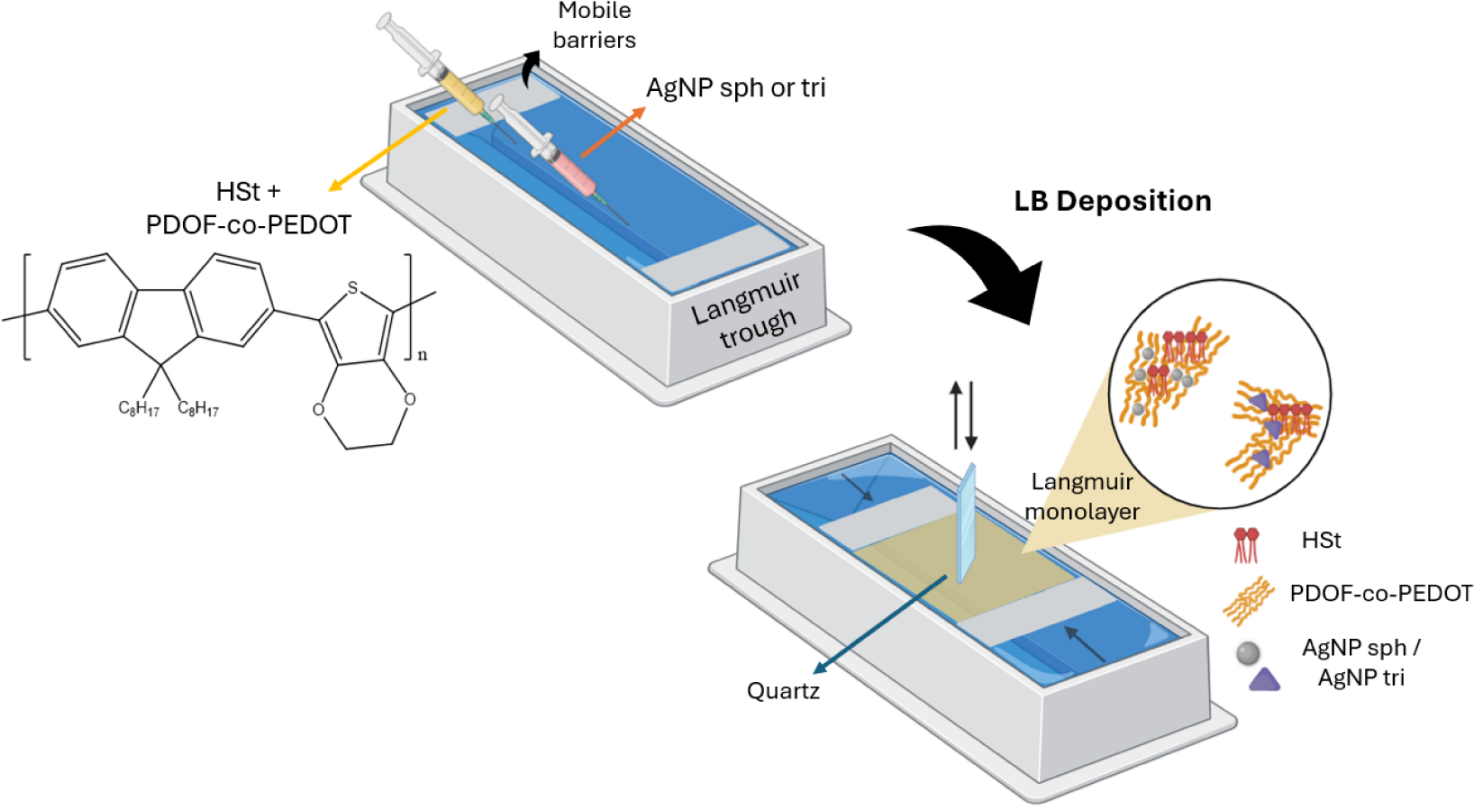
Schematic showing the formation of Langmuir monolayers and LB films of HSt, PDOF-*co*-PEDOT with spherical or triangular AgNP (AgNP sph or AgNP tri).

For dilatational rheology experiments, the monolayers were compressed to 30 mN m^–1^, followed by 10 min of stabilization period to maintain stead pressure with the barriers going back and forth. After stabilization, with the interface not subjected to any significant barrier movement to maintain constant surface pressure, the monolayers underwent 10 compression–expansion cycles with a 1% area variation at a frequency of 20 mHz. The surface dilatational modulus quantifies the resistance of an interface to area deformations and is obtained from the change in surface pressure (π) as a function of a relative change in surface area (*A*).^25^ This parameter characterizes the interfacial rheological response by describing the relationship between the applied surface stress and the resulting surface strain. In oscillatory experiments, the dilatational modulus is a complex quantity that can be separated into two contributions: the elastic modulus (*E*′), associated with energy storage and reversible molecular reorganization at the interface, and the viscous modulus (*E*″), related to energy dissipation and irreversible processes such as molecular diffusion and reorientation eq 1:

1 $$E=\mathrm{-A}\left(\frac{\Delta \pi }{A}\right)=E^{\prime }\cos\theta +iE^{\prime \prime }\sin\theta$$

The elastic modulus (*E*′) and the viscous modulus (*E*″) correspond to the real and imaginary components, respectively, of the complex dilatational modulus. The phase angle, θ, is calculated by the phase difference between the oscillations of surface pressure and area. These parameters reflect the viscoelastic nature of the monolayer. In a purely elastic system, *E*″ approaches zero, indicating that the response is dominated by energy storage. Conversely, in a fully viscous system, *E*′ tends toward zero, reflecting a response governed primarily by energy dissipation.^25–27^

Polarization-modulation infrared reflection–absorption spectroscopy (PM-IRRAS) spectra of Langmuir and LB films were measured with a spectrophotometer KSV PMI 550—KSV Nima Instruments (incidence angle of 80° to the interface normal). Brewster angle microscopy (BAM) images before, during and after compression were taken with the MicroBAM instrument (KSV Nima Instruments) with a fixed angle of 53° and an image resolution of 12 μm. It should be noted that a Langmuir trough (small model—KSV Instruments) with a total subphase volume of 50 mL was used for the isotherms and cycles. For BAM and PM-IRRAS, a Langmuir trough (KSV Instruments) with a total subphase volume of 210 mL was used (medium model—KSV Instruments).

### Preparation of Langmuir–Blodgett (LB) Films

The transfer of the monolayers of Hst (70 μL), HSt:PDOF-*co*-PEDOT (3:1 v/v – 70 μL) or HST:PDOF-*co*-PEDOT with spherical or triangular AgNP (10 μL of AgNP dispersions) onto a solid support (quartz – 1 cm × 2 cm) was carried out at an immersion rate of 5 mm min^–1^ at a constant surface pressure of 30 mN m^–1^. At this surface pressure, the monolayers at the subphase were sufficiently compacted and ordered before collapse. An interval of 10 min was maintained between the upward stroke and the subsequent downward stroke (Figure 1). During the transfer process, the surface pressure was kept constant and the transfer ratio (TR) values were determined to evaluate the successful transfer of the monolayers (TR values close to 1). The LB layers were characterized by PM-IRRAS and Raman spectroscopy. The masses of the deposited materials were determined according to the Sauerbrey equation^28^ using a QCM200 microbalance (Stanford Research Systems).

### Raman Spectroscopy Studies

The Raman spectra of the films were recorded with a Renishaw InVia reflex spectrometer at room temperature and air atmosphere using a He–Ne laser with an excitation line of 632.8 nm, a laser power of 1 mW and an exposure time of 20 s. For the studies with the pesticide CLP, the LB films were immersed in a solution of 10^–4^, 10^–5^ and 6 × 10^–6^ mol L^–1^ CLP (acetonitrile/water −1:1 v/v) for 6 h (optimized time). After this time and drying at local room temperature (25 ± 1 °C) for 10 min, the films were analyzed by Raman spectroscopy under the same specific conditions as described above. For comparison, 5-layer spin-coated films of PDOF-*co*-PEDOT with spherical AgNP were prepared by Spin Coating technique (Microtube—SpinCoater model A1 V1.1.2). Alternating layers of PDOF-*co*-PEDOT and AgNP sph were deposited on quartz (1 cm × 2 cm). The same solutions used for the preparation of the LB films were used for spin-coated films, with deposition parameters of 100 μL and 500 rpm for 40 s. As the LB films, the spin-coated film was immersed in the CLP solution for 6 h before drying and Raman analysis.

## Results and Discussion

### Properties of the Langmuir Monolayers

Before studying the air–water interfacial interactions between the synthesized materials for the development of LB films, the original materials (PDOF-*co*-PEDOT and spherical and triangular AgNP) were properly synthesized and characterized. They exhibit structural features and properties consistent with each material, as reported in our earlier study.^22^

In Langmuir films, polymeric systems are generally more complex than those consisting only of small amphiphilic molecules. In contrast to lipids or fatty acids, which usually have well-defined phase transitions, conjugated polymer chains tend to fold and intercalate during compression, resulting in rigid monolayers with poorly defined phase transitions.^10,26^ As shown in Figure S1A and S1B, the π-*A* isotherms of pure PDOF-*co*-PEDOT interfacial films do not show a clear distinction between the liquid-expanded and condensed phases. Furthermore, the isotherms shift toward smaller molecular areas with increasing fraction of incorporated PDOF-*co*-PEDOT. This trend indicates that the monolayer occupies less interfacial area due to the aggregation of the polymer chains by cohesive interactions, which promotes the formation of 3D structures and limits molecular dispersion at the air–water interface.^26^ The formation of these 3D aggregates occurs during the compression of the monolayer, as shown by the compression–decompression cycles of the pure PDOF-*co*-PEDOT monolayers (Figure S1C). In these cycles, the decompression curves reach lower surface pressures than the corresponding compression curves within the same molecular surface, indicating the occurrence of hysteresis. The irreversible nature of the aggregation process during compression is further confirmed by the progressive shift of the π-*A* isotherms toward smaller regions during repeated cycles, reflecting increasing film condensation. Brewster angle microscopy (BAM) images (Figure S2) support these results, as they show that large polymeric domains formed during compression persist after decompression. These domains exhibit irregular patterns related to folding and entanglement of the polymer chains, consistent with previous reports of Langmuir films of conjugated polymers.^10,29–31^

A common strategy to mitigate strong cohesive forces in polymer-based monolayers is cospreading with fatty acids or lipids.^10^ These amphiphilic molecules form a neutral matrix that improves the dispersion of the polymer chains, increases the stability of the film and facilitates the incorporation of additional functional materials, such as nanoparticles. To explore this approach, mixed monolayers of PDOF-*co*-PEDOT with stearic acid (HSt) were investigated in different compositions, as shown in Figure 2A. Compared to the pure HSt monolayer, which has a collapse pressure of 51.05 mN m^–1^, increasing the proportion of PDOF-*co*-PEDOT results in mixed monolayers with lower collapse pressures (between 49.02 and 40.35 mN m^–1^) and to isotherms that shift toward smaller areas, indicating the formation of more condensed films. At higher HSt concentrations, the polymeric monolayers exhibit more clearly defined phase transitions, with the π-*A* isotherms shifting to larger molecular areas compared to those of mixed films with higher proportions of PDOF-*co*-PEDOT (Figure 2A). Although the addition of HSt improves the uniformity of the polymer monolayers, molecular cohesion between the polymer chains still occurs, as evidenced by the leftward shift of the isotherms, albeit at a less pronounced level than for a monolayer containing only PDOF-*co*-PEDOT. Based on these properties, the 3:1 HSt:PDOF-*co*-PEDOT composition was selected for the subsequent analyzes as it exhibits well-defined phase behavior and achieves a high surface pressure (48.74 mN m^–1^) over a larger interfacial area before collapse.

**Figure 2:**
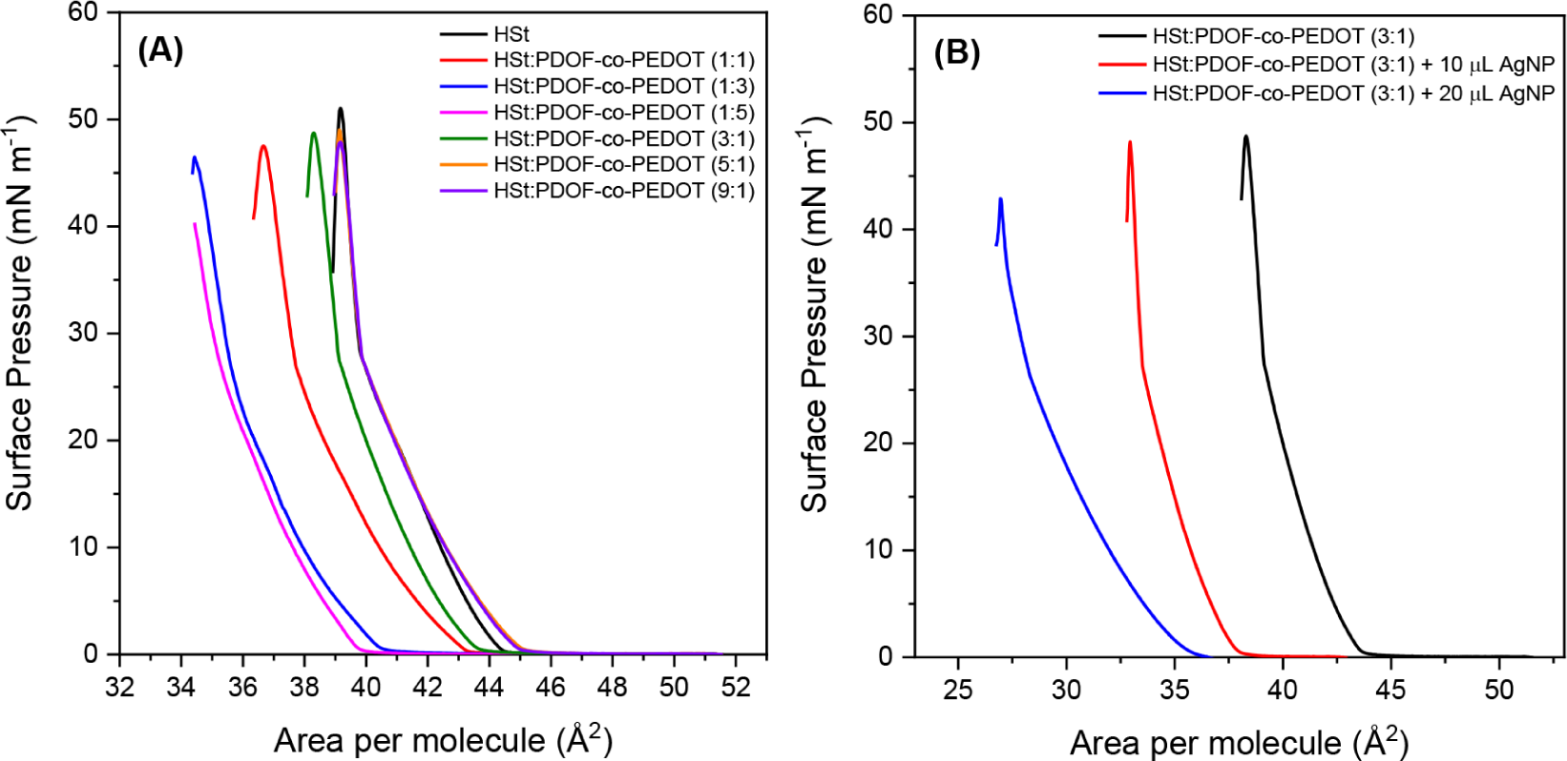
π-*A* isotherms (area per HSt molecule) for (A) different proportions (v/v) of HSt and PDOF-*co*-PEDOT (total volume of 70 mL), concentration solutions of 0.5 mg mL^–1^, using chloroform as solvent; and (B) for different volumes of AgNP sph spread on the PDOF-*co*-PEDOT/HSt monolayer.

Exploratory studies on the incorporation of AgNP in monolayers were first carried out with the spherical (AgNP sph). Figure 2B shows the π-*A* isotherms of HSt:PDOF-*co*-PEDOT films with two different amounts of AgNP sph. Remarkably, the addition of only 10 μL AgNP leads to a clear shift of the isotherm toward smaller molecular areas. This behavior indicates a possible adsorption of AgNP at the interface of HSt and PDOF-*co*-PEDOT. Although the AgNPs are stabilized with sodium citrate, a hydrophilic compound, the spreading of the AgNP dispersion after the spreading of the HSt and PDOF-*co*-PEDOT solution at the subphase promotes their adsorption. The nanoparticles likely occupy the spaces between the polar headgroups of HSt and the aqueous subphase, reducing the lateral electrostatic repulsions.^26^ Furthermore, the characterizations discussed below provide clear evidence for the actual adsorption of AgNP within the monolayer and LB film. When the AgNP volume is increased to 20 μL, the isotherm shows less pronounced phase transitions and a further shift to lower regions, indicating a decrease in molecular packing and spreading at the interface. Furthermore, this film collapses at a lower surface pressure (43 mN m^–1^), suggesting that excessive incorporation of nanoparticles impairs the stability of the mixed monolayer. Based on these observations, the optimized formulation for the subsequent studies consisted of a 3:1 (v/v) HSt:PDOF-*co*-PEDOT mixture (total volume of 70 μL) with the addition of 10 μL AgNP.

The presence of PDOF-*co*-PEDOT in the mixed systems promotes the formation of dispersed aggregates within the subphase or 3D structures extending toward the air, as can be seen in the BAM images (Figure 3 left panels). In contrast to the homogeneous interfacial distribution observed in pure HSt monolayers (Figure S3)—where adhesion forces dominate—the mixed HSt:PDOF-*co*-PEDOT monolayers show the formation and persistence of aggregates during and after compression, indicating that cohesive forces dominate adhesion forces. However, these domains are significantly smaller, less dense and more evenly distributed than in the pure PDOF-*co*-PEDOT monolayers (Figure S2). In addition, the mixed monolayers show improved reversibility upon decompression, with even smaller domains after decompression (Figure 3D left panels), indicating a partial weakening of cohesive interactions and improved dynamic behavior at the interface. The BAM images of the HSt:PDOF-*co*-PEDOT/AgNP sph monolayer (Figure 3, right panels) show how the adsorption of AgNP influences the formation of the domains/agglomerates previously seen in pure PDOF-*co*-PEDOT and HSt:PDOF-*co*-PEDOT monolayers. The compression even leads to the formation of smaller and more homogeneously distributed domains (Figures 3B and 2C right panels), which proves the adsorption of AgNP in the mixed monolayer. The same behavior was observed for the mixed film with triangular nanoparticles (AgNP tri), as shown in Figure S4.

**Figure 3:**
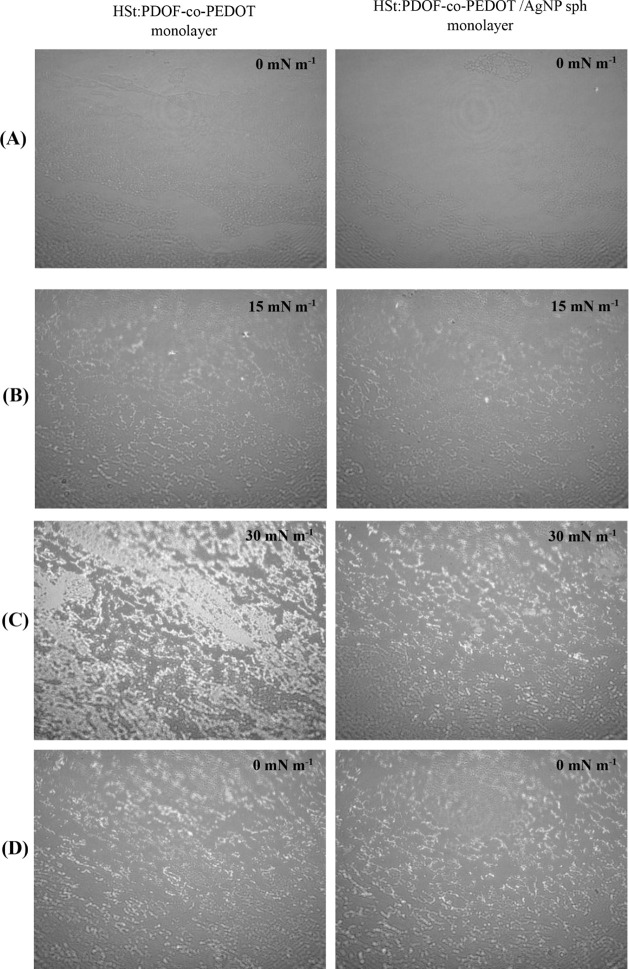
BAM images (3600 × 4000 mm) of HSt:PDOF-*co*-PEDOT (left panels) and HSt:PDOF-*co*-PEDOT/AgNP sph (right panels) monolayers, (A) before and (B) during compression, (C) compressed and (D) after decompression.

Figure 4A–C summarizes the investigations of the π-*A* and Δ*V*-*A* isotherms and the surface compression modulus of optimized HSt:PDOF-*co*-PEDOT monolayers, both without and with AgNP sph or AgNP tri. As previously observed, the addition of AgNP shifts the π-*A* isotherms to lower ranges, especially in the case of AgNP sph (Figure 4A—red curve), indicating different molecular reorganizations of the HSt:PDOF-*co*-PEDOT monolayer for each studied morphology of AgNP. The morphology of the particles exhibits different surface energies, which directly affect their properties, including adsorption capacity.^16,32,33^ The AgNP tri synthesized in this work are larger than AgNP sph, measuring 29 nm compared to 13 nm for the spherical ones.^22^ Possibly, due to their smaller size and spherical shape, the AgNP exhibit more efficient and homogeneous adsorption in the monolayer, penetrating the conducting polymer chains and promoting better molecular restructuring. Moreover, since they are isotropic, they do not have a preferred orientation that could limit their interaction with the monolayer. In contrast, the larger size and the triangular shape of AgNP tri could limit their adsorption between HSt and PDOF co-PEDOT molecules due to their higher structural rigidity and the resulting preferential orientation in the molecular organization.

**Figure 4:**
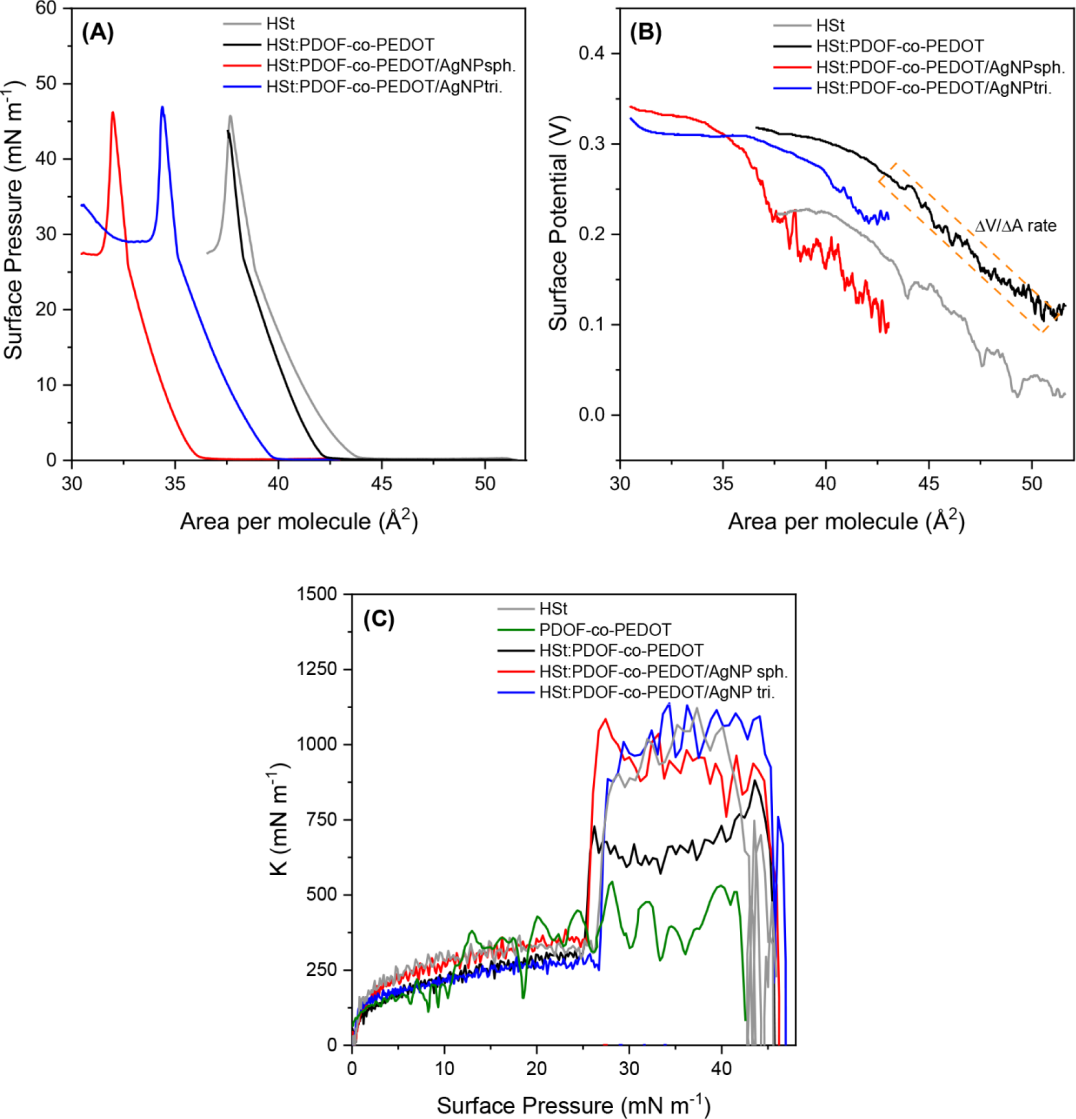
(A) π-*A* and (B) Δ*V*-*A* isotherms (area per HSt molecule) for monolayers of neat HSt, HSt:PDOF-*co*-PEDOT and HST:PDOF-*co*-PEDOT with AgNP sph or AgNP tri; and (C) compressional modulus (*K*) as a function of surface pressure of HSt:PDOF-*co*-PEDOT with AgNP sph or AgNP tri.

In agreement with the results of the π-*A* isotherms, the Δ*V*-*A* isotherms (Figure 4B) confirm that the presence of PDOF-*co*-PEDOT in the monolayers leads to the formation of aggregates/domains that are vertically oriented within the monolayer even before compression begins. This is evidenced by the potential isotherm of the mixed monolayers, which starts at higher potential values compared to the pure HSt monolayer. It is noteworthy that the addition of PDOF-*co*-PEDOT to the monolayers did not lead to a decrease in the maximum potential values, which were kept at around 300 mV as in the pure HSt monolayer. This indicates a random orientation effect of the electric dipoles of the polymer chains, maintaining some orientation of the monolayer, and the absence of a hydration effect on the polar groups of HSt, as also observed in the PM-IRRAS results discussed later. When both types of AgNP are added, the increase in potential values occurs at a higher ΔV/ΔA ratio than for monolayers without AgNP. This suggests that films containing AgNP provide fewer opportunities for organizational conformations when the monolayer is compressed. Interestingly, this effect is more pronounced in the monolayer with AgNP tri, which has a higher ΔV/ΔA ratio, indicating that the triangular shape and larger size of these nanoparticles act as limiting factors in the molecular reorganization of the monolayer.

Figure 4C shows the surface compression modulus (K) of the pure and mixed monolayers. Derived from the π-*A* isotherms using the equation $K=-A{\left(\frac{\partial \pi }{\partial A}\right)}_{T}$ ,^34^ these values are related to the in-plane elasticity of the Langmuir films and indicate the resistance of the monolayer to changes in molecular area during compression. Higher *K* values therefore correspond to a more compact monolayer that is sensitive to compression, while lower values indicate a more fluid and disordered state of the monolayer.^35^ The addition of PDOF-*co*-PEDOT to the HSt monolayer leads to a reduction in *K* values, which nevertheless remain higher than those of the pure PDOF-*co*-PEDOT monolayer. This indicates that the presence of HSt improves the organization of the polymer monolayer. At both lower and higher surface pressures, corresponding to the liquid condensed state (100–250 mN m^–1^) and the solid state (>500 mN m^–1^), respectively,^26^ the copolymer disrupts the highly ordered packing of the HSt molecules, making the monolayer more fluid and prone to molecular rearrangements. Regardless of its form, the addition of AgNP leads to higher K values (stiffer monolayers), which is due to the high condensation state of the supramolecular structure formed from the three components.

The compression modulus (K) is a thermodynamic property and represents the purely elastic response of a monolayer when subjected to lateral compression (quasi-static). When another mechanical perturbation is applied—such as dynamic measurements with compression–expansion cycles over time from a predetermined surface pressure, different values can be obtained and both the elastic and viscous properties of the monolayer can be evaluated.^26,35,36^ On this basis, the viscoelastic behavior of monolayers of HSt and PDOF-*co*-PEDOT, with and without AgNP sph, was investigated (Table 1). Only the spherical nanoparticles were considered, as no significant differences in the *K* values were observed between the different AgNP forms.

**Table 1:** Rheological Data for Pure and Mixed Monolayers Compressed to 30 mn m^–1^ and Subjected to 10 Cycles of Compression-Expansion (1% of Area Variation and Frequency of 20 mhz)^a^

Monolayer	*E* (mN m^–1^)	*E*’ (mN m^–1^)	*E*” (mN m^–1^)	θ (rad)
HSt	137.23	132.41	36.06	0.27
HSt:PDOF-*co*-PEDOT	169.64	164.71	40.60	0.24
HSt:PDOF-*co*-PEDOT/AgNP sph.	149.28	144.82	36.21	0.25

a Each value is an average of three measurements, with a standard deviation lower than 5%.

First, it can be observed that the addition of PDOF-*co*-PEDOT, followed by AgNP sph, to the HSt monolayer does not significantly alter the balance between the elastic (*E*’) and viscous (*E*”) contributions, as the phase angle values (θ) remain largely unchanged. Interestingly, the inclusion of PDOF-*co*-PEDOT in the HSt monolayer leads to an increase in elasticity—as evidenced by higher *E* and *E*’ values—which contrasts with the behavior observed for the K values, where a decrease was observed. It is worth noting that the short compression/decompression cycles used in the dynamic dilation modulus (*E*) analysis, as opposed to the continuous compression process used to determine the *K* values, promote relaxation of the monolayer. This more stable molecular arrangement allows the monolayer to respond more elastically to cyclic stimuli while exhibiting greater structural rigidity. The subsequent addition of AgNP sph restores the fluidity of the HSt:PDF-*co*-PEDOT monolayer, as shown by the decrease in elasticity. This indicates that AgNP disrupts the tight molecular packing between HSt and PDOF-*co*-PEDOT, making the monolayer more fluid and susceptible to structural rearrangements under this type of mechanical loading.

The monolayers were characterized by PM-IRRAS at a surface pressure of 30 mN m^–1^ (Figure 5A and 5B), a transfer pressure at which the Langmuir monolayer is sufficiently compacted and ordered before the collapse or total compression of the film, as analyzed in the isotherms.

**Figure 5:**
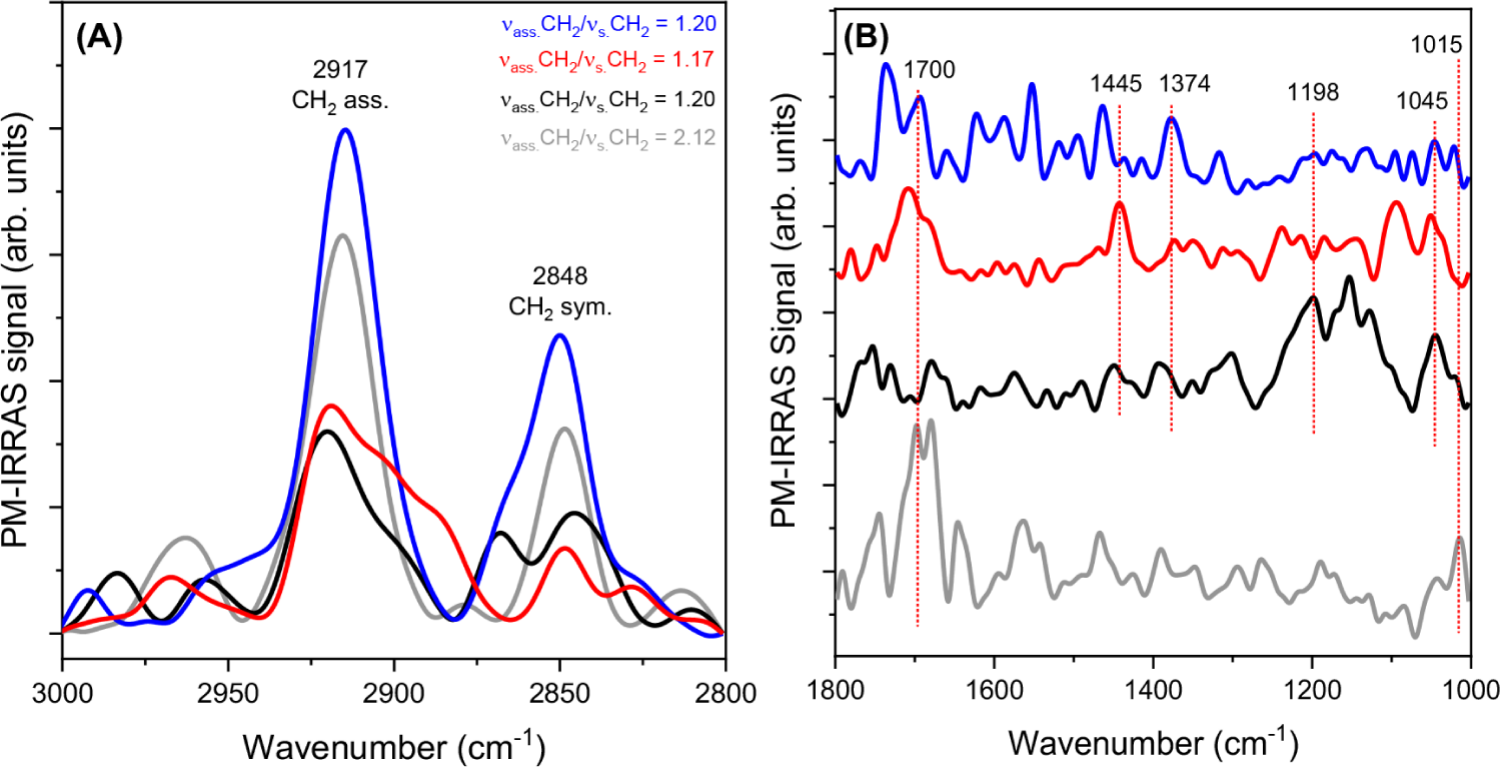
PM-IRRAS spectra of neat HSt (gray spectra), HSt:PDOF-*co*-PEDOT (black spectra), HSt:PDOF-*co*-PEDOT/AgNP sph (red spectra) and HSt:PDOF-*co*-PEDOT/AgNP tri (blue spectra) monolayers (at 30 mN m^–1^) in the (A) 3000–2800 cm^–1^ and (B) 1800–1000 cm^–1^ regions. Figure A shows the order parameters related to HSt bands.

In the region between 2800 and 3000 cm^–1^ (Figure 5A), bands at 2848 and 2917 cm^–1^ are observed, which are attributed to the symmetric and asymmetric CH_2_ stretching vibrations, respectively, associated with the hydrophobic regions of HSt.^37,38^ The ratio between the intensities of these two bands, known as the order parameter, is directly related to the degree of disorder in the nonpolar groups of HSt.^39,40^ Compared to the pure HSt monolayer, the addition of PDOF-*co*-PEDOT and AgNP decreases the order parameter (inset Figure 5A), and the effect is even more pronounced in the monolayer with AgNP sph. This indicates a reduction in the ratio between trans and gauche conformers and a distortion of the acyl chains.^37^

Figure 5B highlights the region between 1000 and 1800 cm^–1^ where characteristic bands of both HSt and PDOF-*co*-PEDOT can be seen. For the mixed monolayers with PDOF-*co*-PEDOT, the bands at 1045, 1198, 1374, and 1445 cm^–1^ correspond to the asymmetric C–O–C stretching of the EDOT group, the C=C and C–C stretching of the thiophene rings, the C–S–C stretching of the EDOT group and the asymmetric C–H (CH_3_) deformation, respectively.^30,41,42^ The bands at 1015 and 1700 cm^–1^ are attributed to the vibrations of the phosphate group and the C=O stretching of the carbonyl group in HSt, respectively.^26,36^ For the monolayers containing PDOF-*co*-PEDOT and AgNP, a shift of the 1700 cm^–1^ band to higher wavenumbers is observed. This band is associated with the hydration of the carbonyl groups in HSt, suggesting that the presence of PDOF-*co*-PEDOT and AgNP reduces the degree of hydration of these groups within the monolayer.^43,44^

### Transfer of Langmuir Monolayers to Solid Support

After characterization of the optimized Langmuir monolayers, LB films were prepared and analyzed. During the formation of an LB film, the control of the transfer of the material on the substrate is evaluated by the transfer rate (TR), which is defined as the ratio between the decrease in the area of the Langmuir monolayer (to be transferred) and the immersed area of the substrate. A TR value close to 1 indicates a more efficient and uniform substrate coverage. However, due to molecular rearrangements and the inherent complexity of the system, TR values greater or less than 1 may also occur. Negative TR values can also be observed, indicating a loss of material into the subphase during the immersion or emersion processes.^26^ TR values of up to 0.55 were obtained, with more efficient transfer (i.e., higher TR values) occurring during the substrate emersion steps (odd numbers), as shown in Figure S5A. Although the TR values are relatively low, they are in line with expectations as systems with polymers and metallic nanoparticles tend to form stiffer interfacial films that can hinder transfer.^3,26^ TR values that deviate from 1.0 for polymer films may be related to chain entanglement and folding/unfolding processes within the monolayer, which can lead to uneven adhesion of the polymer to either the solid substrate or the previously transferred layer.^10,26,30^ A linear growth in the amount of material deposited was also observed, as seen in Figure S5A, which shows the cumulative TR values as a function of the number of layers deposited. These results indicate the formation of Y-type LB films, where transfer occurs both when the substrate is immersed and when it emerges. This linear transfer could be verified using a quartz crystal microbalance, as shown in Figure S5B. Both the pure HSt LB film and the mixed films containing PDOF-*co*-PEDOT and AgNP showed a linear increase in mass with the number of layers deposited. In particular, the incorporation of additional components, such as the copolymer and the metallic nanoparticles, led to higher mass values for the same number of layers, indicating an effective contribution of these materials to the overall structure of the film. This behavior confirms the successful incorporation of PDOF-*co*-PEDOT and spherical or triangular AgNP into the LB films.

Figures 6 and S6 show the PM IRRAS spectra of the mixed LB films, with and without AgNP, and the pure HSt LB film. Compared to the monolayers (Figure 5), the shift of the CH_2_ stretching bands to lower wavenumbers (from 2917 to 2909 cm^–1^) and the increase of the order parameters show a more compact and oriented organization in the LB films, both in the pure HSt film and in the mixed films. In other words, the LB films exhibit a higher degree of order in the all-trans conformations of the alkyl chains and a lower number of Gauche defects.^45,46^

**Figure 6:**
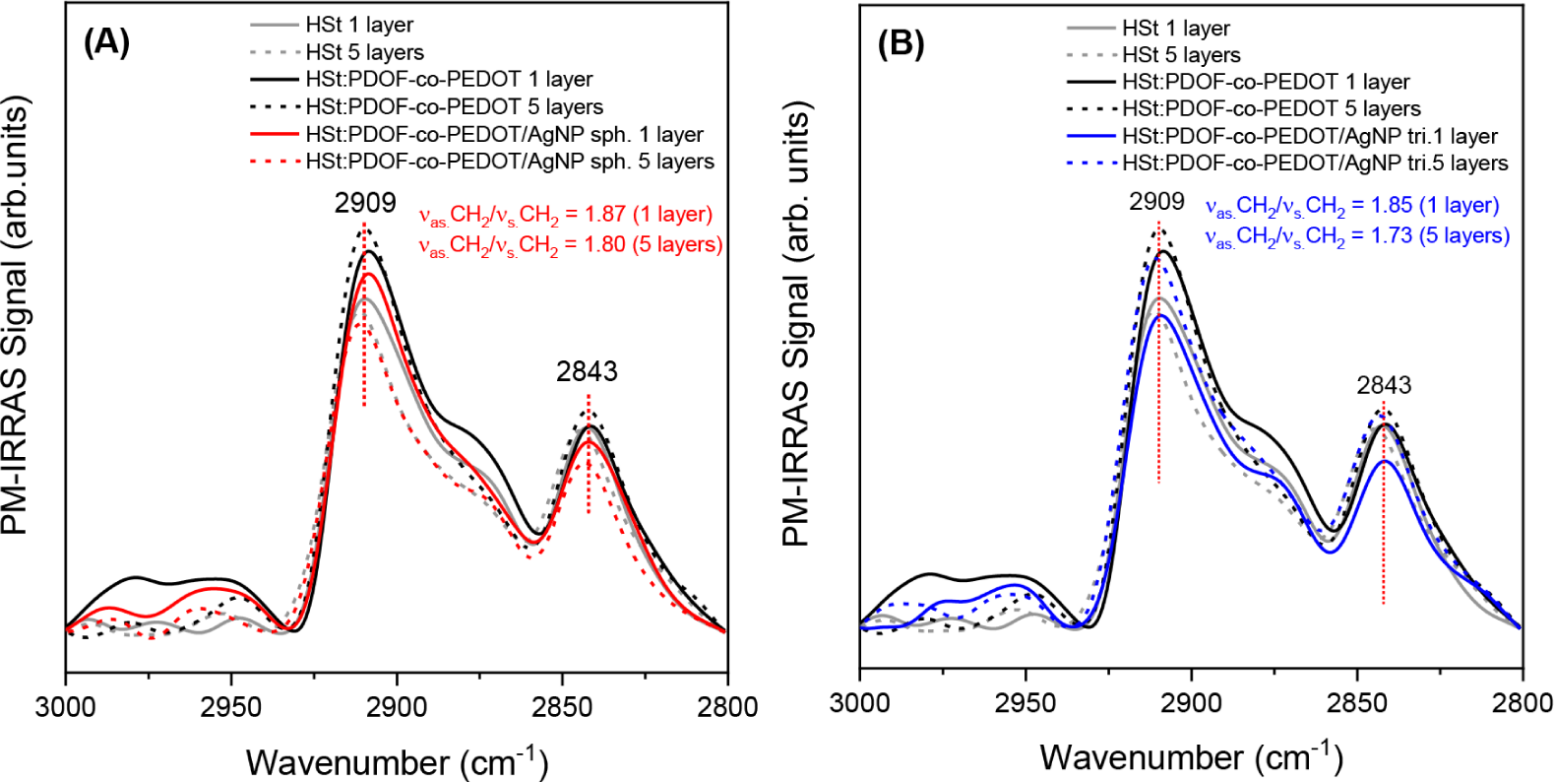
PM-IRRAS spectra of neat HSt and HSt:PDOF-*co*-PEDOT LB films compared to (A) HSt:PDOF-*co*-PEDOT/AgNP sph and (B) HSt:PDOF-*co*-PEDOT/AgNP tri films, and respective order parameters.

Notably, the incorporation of PDOF-*co*-PEDOT induces molecular organization during the transfer, as evidenced by the higher order parameter values in the LB films containing the copolymer compared to those of the pure HSt film (Figure S6). Furthermore, the presence of AgNP tri appears to have a negative effect on monolayer transfer, as lower order parameter values were observed in the films with AgNP tri compared to those with AgNP sph. Therefore, in addition to limiting the molecular reorganization of the monolayer previously observed in the isothermal studies, the presence of AgNP tri leads to LB films with a lower degree of structural organization and a lower amount of deposited material, as shown by the lower value of the deposited weight (Figure S5A) for the film with AgNP tri.

The morphology and optical properties of the LB films without and with spherical AgNP (the latter corresponding to the film with a higher amount of deposited material and a higher degree of organization) were analyzed, as shown in Figures S7 and S8. As expected, the SEM images (Figure S7) showed that the LB films were homogeneous, and this homogeneity was maintained even after the incorporation of AgNP sph. For comparison, a PDOF-*co*-PEDOT film containing AgNP sph was also prepared using the spin coating technique, which showed less homogeneity of the deposited material on the substrate (Figure S7C).

In terms of optical properties, the, LB films exhibited less pronounced emission bands regardless of the presence of AgNP (Figure S8A), with the emission maximum being red-shifted to higher wavelengths (from 456 nm to 482/494 nm) compared to PDOF-*co*-PEDOT in solution. This behavior is characteristic of the formation of excimers, i.e., molecular complexes that are usually formed in systems containing solid conjugated polymers (films).^47,48^ It is noteworthy that the bathochromic shift is more pronounced in the film containing AgNP, which also exhibits a higher emission intensity. The enhancement of the photoluminescence of conjugated polymers by metallic nanoparticles can be attributed to the strong influence of the surface plasmon resonance (SPR) effect of metallic nanoparticles (near-field effect).^49,50^ Upon excitation, the AgNPs in the films generate an intense and localized electromagnetic field. Due to their proximity to the PDOF-*co*-PEDOT, this field enhances the emission of the copolymer.^49–51^ Evidence of this effect is provided by the overlap between the absorption spectra (Figure S8B) of PDOF-*co*-PEDOT (λ_max abs_ = 383 nm) and the AgNP (λ_max abs_ = 390 nm). Such overlap indicates that the plasmonic energy of the AgNP can be transferred to the nearby PDOF-*co*-PEDOT, enhancing their optical properties.^50^

### Studies in Raman Spectroscopy: Potential for SERS Sensing

As shown, highly organized films containing PDOF-*co*-PEDOT and AgNP were successfully prepared using the Langmuir–Blodgett (LB) technique. As previously mentioned, the high degree of control over molecular organization and transfer provided by the LB technique can be a key factor in optimizing SERS sensors, as it allows for a more homogeneous AgNP distribution and the formation of more hotspot regions. Therefore, the LB films were investigated for the detection of the pesticide chlorpyrifos (CLP) by immersing these films in solutions with different concentrations of CLP and then analyzing the SERS signals characteristic of the target molecule, as shown in Figure 7 for the LB films of HSt:PDOF-*co*-PEDOT/AgNP sph. In contrast to the films with spherical AgNP, films prepared with triangular nanoparticles showed limitations in the reproducibility of the SERS spectra, highlighting the influence of the geometry of the plasmonic nanoparticles on the SERS effect and consequently on the efficiency of CLP detection. For spherical nanoparticles, the hotspots are mainly located at the junctions between the particles, making the control of particle aggregation a key factor for the concentration of these spots.^17,52^ On the other hand, anisotropic nanoparticles—such as triangular ones—concentrate hotspots at their tips and edges without the need for controlled aggregation, but they are thermodynamically less stable.^52^ This reduced stability compromises the system’s reproducibility, as observed in the AgNP tri films studied here. In addition, AgNP tri was found to hinder the molecular reorganization of the floating monolayers, resulting in LB films with reduced material transfer and less compact, less ordered structures. This diminished structural order may be a key factor limiting SERS enhancement and the overall performance of these active substrates

**Figure 7:**
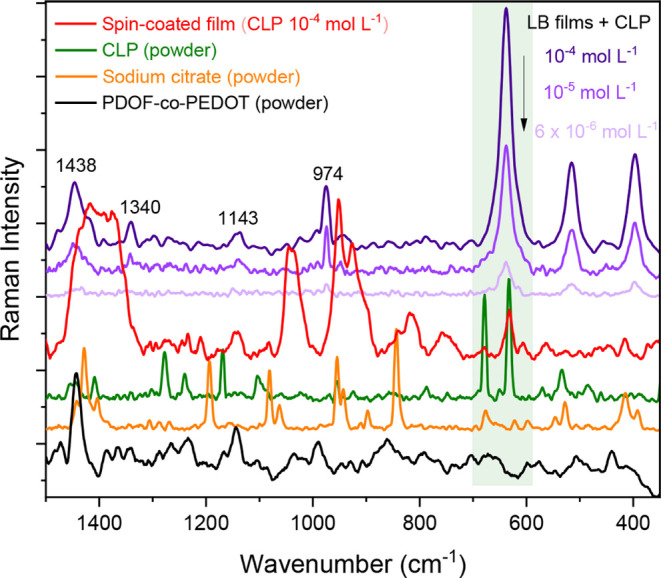
Raman spectra of 5-layer films of HSt:PDOF-*co*-PEDOT/AgNP sph. after immersion in solutions with different concentrations of CLP (purple spectra) in comparison with Raman spectra of 5-layer spin-coated film of PDOF-*co*-PEDOT/AgNP sph (red spectrum), neat CLP (green spectrum), sodium citrate (orange spectrum) and PDOF-*co*-PEDOT (solid-black spectrum).

Figure 7 shows the Raman spectra of the 5-layer LB films of HSt:PDOF-*co*-PEDOT/AgNP sph after 6 h of immersion in solutions containing three different concentrations of CLP. This immersion time was selected to ensure reliable acquisition of the Raman signal. Characteristic bands of PDOF-*co*-PEDOT can be observed in the LB films, e.g., at 1438, 1340, and 1143 cm^–1^, which correspond to the symmetric Cα=Cβ and Cβ–Cβ stretching of the thiophene ring and the C–H deformation of the fluorene side chains, respectively (Table 2). Due to the presence of the stabilizing agent sodium citrate, which is used in the preparation of AgNP, characteristic bands of this compound were also observed. In particular, the shift of these bands, such as the one at 974 cm^–1^, indicates an interaction between the citrate and the metallic surface of AgNP.^53,54^

**Table 2:** Assignments of Raman Signals of PDOF-*co*-PEDOT, Sodium Citrate and Chlorpyrifos (CLP)

Raman Signal (cm^–1^)		Assignments	Literature
PDOF-*co*-PEDOT	1438	υ s. Cα=Cβ of thiophene	^59^
	1340	υ Cβ -Cβ of thiophene ring	^59,60^
	1264	δC–H of polymeric chain	^61^
	1143	δ C–H of fluorene lateral chains	^61^
Sodium citrate	1428	υ C–O + δ O–H + υ s. CO_2_	^53^
	1192	υ C–C–O	^53^
	1081	υ C–O	^53,62^
	954	υ C–C–O_2_	^53,59^ ,^62^
	844	υ s. CCCC-O	^53,62^
CLP	678	δC–Cl	^63,64^
	633	υ C–Cl and υ *p* = S	^63,64^
	532	υ P–O	^63,64^

Compared to the films obtained by spin-coating (pink curve - Figure 7), the LB films showed more intense and clearer Raman signals for CLP (633 cm^–1^), even at low analyte concentrations (10^–5^ and 6 × 10^–6^ mol L^–1^), indicating a greater efficiency in amplifying the Raman signals. This improvement can be attributed to the ability of the LB technique to organize both the copolymer and AgNP in a more ordered and controlled manner at the air/water interface, resulting in a higher density of hotspots that are important for SERS amplification.^12,20,21^ In addition, the presence of the conjugated polymer can promote π-π and electrostatic interactions with the CLP, contributing to signal selectivity and intensification.^55^

Despite the promising results, an important limitation was the low reproducibility of the SERS signals at lower CLP concentrations. This variability is attributed to the sensitivity of the LB technique to small variations in the transfer parameters such as immersion velocity, surface pressure and dispersion solution concentration.^13^ These results emphasize the dual nature of the LB technique in the preparation of SERS substrates: On the one hand, it provides a high degree of molecular control and nanoparticle organization, which contributes to improved Raman signal intensity and spectral clarity. On the other hand, it also poses a challenge in terms of reproducibility and stability, especially when working with anisotropic or less stable nanostructures.

It is worth mentioning that, to our knowledge, there are very few reports in the literature describing SERS platforms based on conjugated polymers or silver nanoparticles constructed using the LB technique. Most of the existing work focuses on isolated metal nanoparticles or combinations with surfactants, while the incorporation of electronically active copolymers as functional matrix is still poorly explored. In this context, the use of an electroactive polymer such as PDOF-*co*-PEDOT as an organizing and functional matrix within a structured LB architecture represents an innovative and promising strategy. Although the detection limits achieved here are not the lowest that have been reported^56–58^ the combination of structural organization, molecular interaction potential, and optical/electronic functionalities offers a distinct advantage in terms of substrate tunability, film homogeneity, and future application in trace impurity detection. This work therefore contributes to a new branch of research by demonstrating the potential of combining organized LB films and electronically active organic materials for the development of versatile platforms for environmental monitoring.

## Conclusions

This study demonstrates the fabrication of nanostructured Langmuir–Blodgett films combining the conjugated copolymer PDOF-*co*-PEDOT with silver nanoparticles (AgNP) of different shapes. The technique allowed precise control of molecular organization and particle distribution at the air–water interface, resulting in compact and ordered multilayer films. The results highlight the importance of the interactions between conjugated polymers and metal nanoparticles in the fabrication of nanostructured films for SERS sensing. The stable interfacial films formed even with spherical and triangular AgNPs emphasize the potential of hybrid materials to control molecular ordering. PM-IRRAS and spectroscopic analyzes emphasize the key role of nanoparticle geometry in modulating film properties and open new avenues for the development of functional materials. Anisotropic nanoparticles (AgNP tri) can limit the molecular reorganization of the monolayers and thus influence the amount of deposited material and the degree of film organization. Polymeric LB films with spherical AgNPs showed superior SERS performance for the detection of chlorpyrifos, highlighting the influence of geometry and film structure on Raman signal enhancement. Despite some reproducibility issues with triangular particles, the results confirm the potential of LB films as SERS substrates. This work also provides insight into the under-explored synergies between conjugated polymers, metallic nanoparticles and the LB technique for chemical sensing, especially for SERS sensors. Future efforts will aim to optimize both the transfer process and the immersion time of LB films in CLP solutions, and explore their broader potential for environmental sensing applications.

## Supplementary Material



## Notes

Figure S1: (A) surface pressure as a function of area per polymeric unit for different volumes of PDOF-*co*-PEDOT (0.5 mg mL^–1^), (B) surface pressure as a function of monolayer compression for pure PDOF-*co*-PEDOT monolayers, where 0% represents a fully decompressed monolayer and 100% a fully compressed monolayer, and (C) compression (solid curves) and decompression (dashed curves) cycles of neat PDOF-*co*-PEDOT monolayer (PDOF-*co*-PEDOT volume of 70 mL). Figure S2: BAM images (3600 × 4000 mm) of neat PDOF-*co*-PEDOT monolayer (A) before and (B) during compression, (C) compressed and (D) after decompression. Figure S3: BAM images (3600 × 4000 mm) of neat HSt mon. Figure S4: BAM images (3600 × 4000 mm) of HSt:PDOF-*co*-PEDOT/AgNP_tri_ monolayers (A) before and (B) during compression, (C) compressed and (D) after decompression. Figure S5: (A) TR values as a function of number of layers for LB films of HSt:PDOF-*co*-PEDOT and HSt:PDOF-*co*-PEDOT/AgNP sph and HSt:PDOF-*co*-PEDOT/AgNPtri. Inset: added values of TR as a function of deposited layers for the LB films and (B) total weight deposited as a function of deposited layers (1, 3, 5, 7, and 9 layers) for the films. Figure S6: PM-IRRAS spectra of neat HSt and HSt:PDOF-*co*-PEDOT LB films and respective order parameters. Figure S7: SEM images of 5-layer LB films of HSt:PDOF-*co*-PEDOT and HSt:PDOF-*co*-PEDOT/AgNP sph (A and B, respectively), compared to a 5-layer spin-coated film of PDOF-*co*-PEDOT/AgNP sph (C). Figure S8: Emission (A) and absorption (B) spectra of LB films compared to normalized spectra (dashed curves) of PDOF-*co*-PEDOT (chloroform solution—6 mg L^–1^) and AgNP sph. (aqueous dispersion—60 mg L^–1^) (PDF)
