# Supplementary material for: Langmuir–Blodgett Films of Conjugated Polymer and Silver Nanoparticles: A Possible Substrate for Pesticide SERS-Based Detection
Source: Langmuir. 2025 Sep 9;41(37):25729–41. doi: 10.1021/acs.langmuir.5c03648 (PMC12461926; doi:10.1021/acs.langmuir.5c03648)
Supplement: Supplementary file 1 [file la5c03648_si_001.pdf]

Langmuir–Blodgett Films of Conjugated Polymer and Silver Nanoparticles: A Possible Substrate for Pesticide SERS-Based Detection

Rebeca da Rocha Rodrigues[*1], *Diogo Silva Pellosi*[2], *Luciano Caseli*[1], *Laura Oliveira Péres*[1]

[1]Federal University of São Paulo, Laboratory of Hybrid Materials, Diadema, 09913-030, São Paulo – Brazil

[2]Federal University of Paraná, Macromolecules and Interfaces Research Group, Curitiba, 81531-980, Paraná – Brazil

*Corresponding author: rebeca.rodrigues@unifesp.br

Supporting Information

Figure S1. (A) Surface pressure as a function of area per polymeric unit for different volumes of PDOF-co-PEDOT (0.5 mg mL^{-1}); (B) Surface pressure as a function of monolayer compression for pure PDOF-co-PEDOT monolayers, where 0% represents a fully decompressed monolayer and 100% a fully compressed monolayer, and (C) Compression (solid curves) and decompression (dashed curves) cycles of neat PDOF-co-PEDOT monolayer (PDOF-co-PEDOT volume of 70 mL).

Figure S2. BAM images (3600 × 4000 mm) of neat PDOF-co-PEDOT monolayer (A) before and (B) during compression, (C) compressed and (D) after decompression.

Figure S3. BAM images (3600 × 4000 mm) of neat HSt monolayer (A) before and (B) during compression, (C) compressed and (D) after decompression.

Figure S4. BAM images (3600 × 4000 mm) of HSt:PDOF-co-PEDOT/$AgNP_{tri}$ monolayers (A) before and (B) during compression, (C) compressed and (D) after decompression.

Figure S5. (A) TR values as a function of number of layers for LB films of HSt:PDOF-co-PEDOT and HSt:PDOF-co-PEDOT/AgNP sph. and HSt:PDOF-co-PEDOT/AgNPtri. Inset: added values of TR as a function of deposited layers for the LB films; and (B) total weight deposited as a function of deposited layers (1, 3, 5, 7 and 9 layers) for the films.

Figure S6. PM-IRRAS spectra of neat HSt and HSt:PDOF-co-PEDOT LB films, and respective order parameters.

Figure S7. SEM images of 5-layers LB films of HSt:PDOF-co-PEDOT and HSt:PDOF-co-PEDOT/AgNP sph (A and B, respectively), compared to a 5-layers spin-coated film of PDOF-co-PEDOT/AgNP sph (C).

Figure S8. Emission (A) and absorption (B) spectra of LB films compared to normalized spectra (dashed curves) of PDOF-co-PEDOT (chloroform solution – 6 mg L^{-1}) and AgNP sph. (aqueous dispersion – 60 mg L^{-1}).

Supporting Information

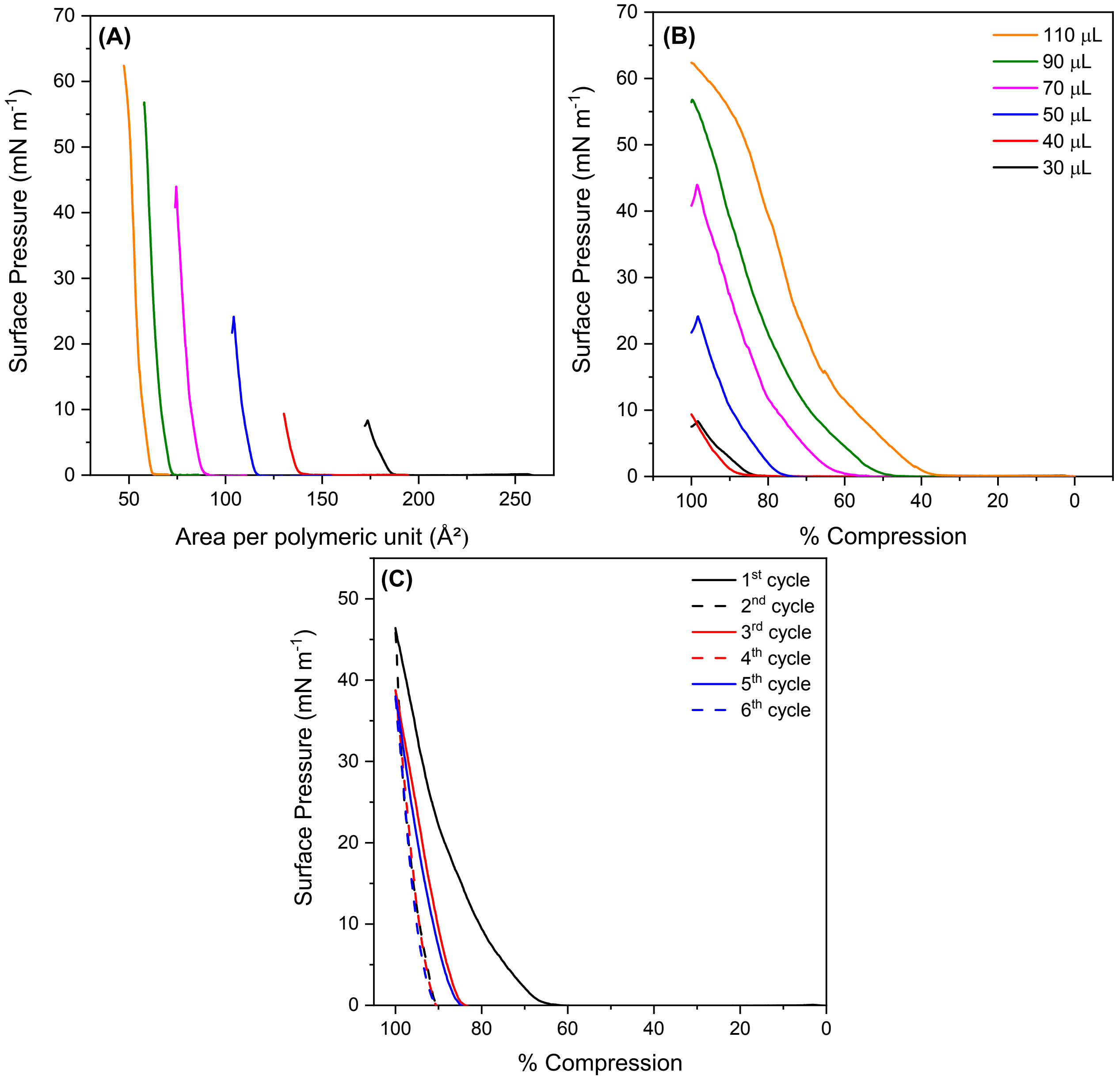

Figure S1. (A) Surface pressure as a function of area per polymeric unit for different volumes of PDOF-co-PEDOT (0.5 mg mL^{-1}); (B) Surface pressure as a function of monolayer compression for pure PDOF-co-PEDOT monolayers, where 0% represents a fully decompressed monolayer and 100% a fully compressed monolayer, and (C) Compression (solid curves) and decompression (dashed curves) cycles of neat PDOF-co-PEDOT monolayer (PDOF-co-PEDOT volume of 70 μL).

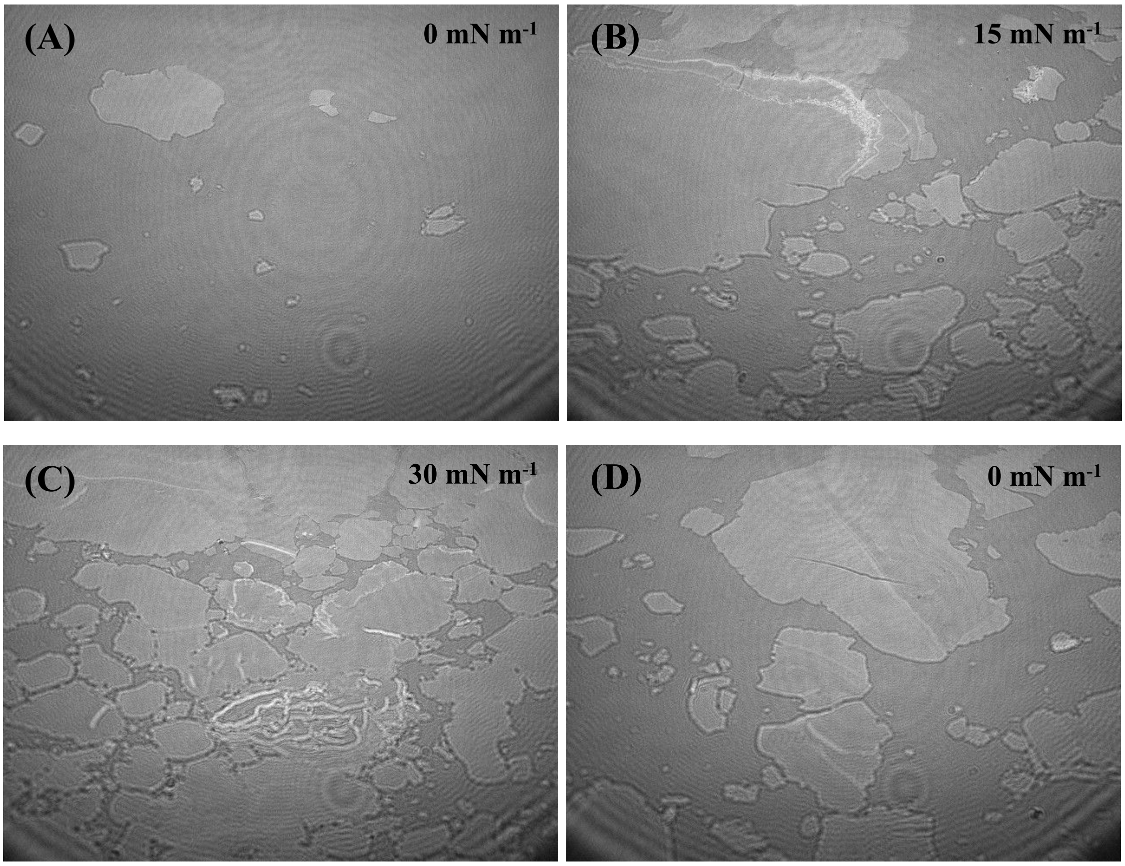

Figure S2. BAM images (3600 × 4000 μm) of neat PDOF-co-PEDOT monolayer (A) before and (B) during compression, (C) compressed and (D) after decompression.

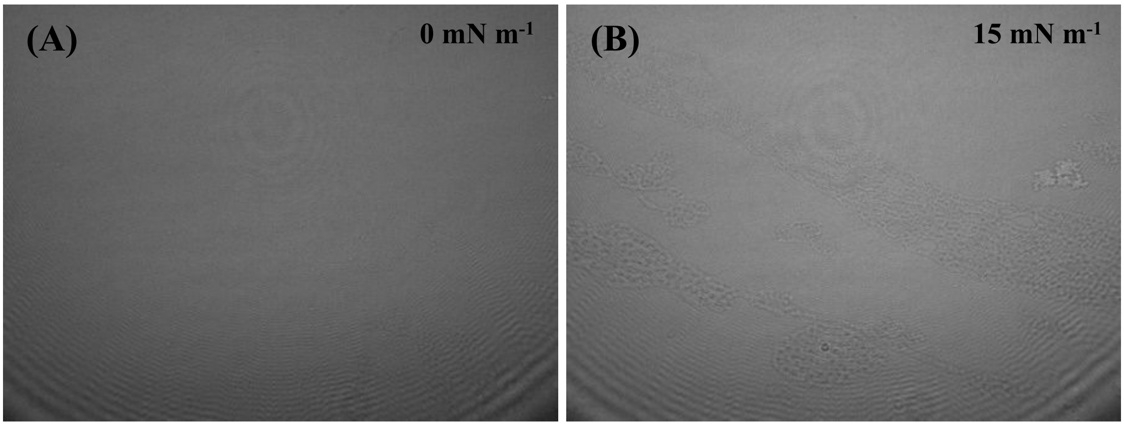

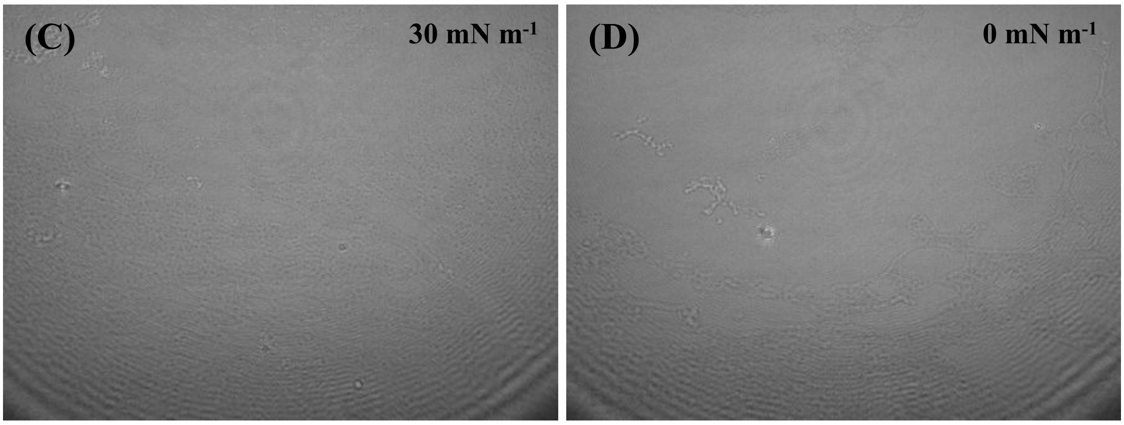

Figure S3. BAM images (3600 × 4000 μm) of neat HSt monolayer (A) before and (B) during compression, (C) compressed and (D) after decompression.

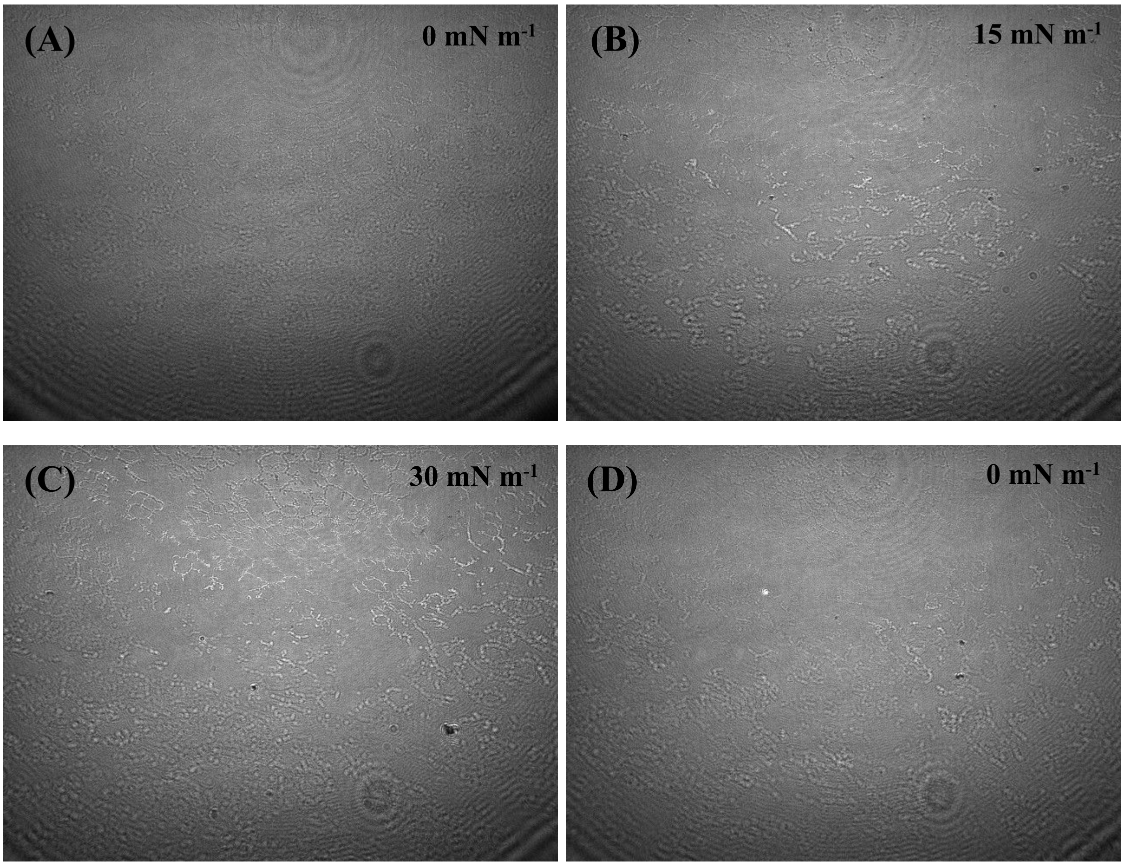

Figure S4. BAM images (3600 × 4000 μm) of HSt:PDOF-co-PEDOT/$AgNP_{tri}$ monolayers (A) before and (B) during compression, (C) compressed and (D) after decompression.

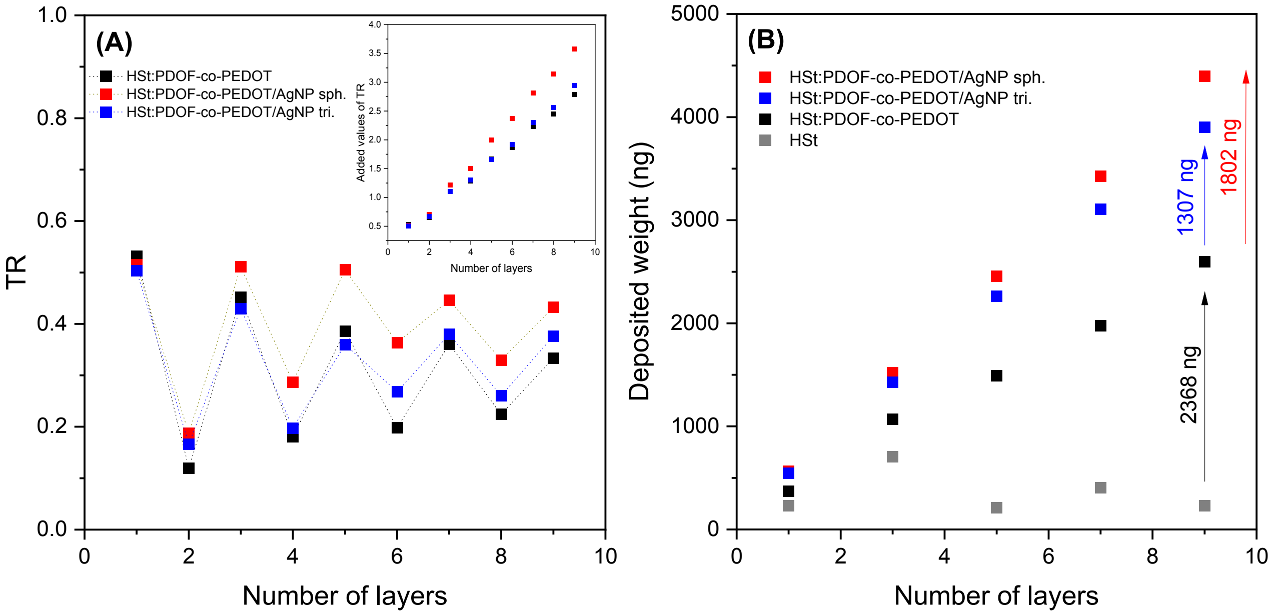

Figure S5. (A) TR values as a function of number of layers for LB films of HSt:PDOF-co-PEDOT and HSt:PDOF-co-PEDOT/AgNP sph. and HSt:PDOF-co-PEDOT/AgNPtri. Inset: added values of TR as a function of deposited layers for the LB films; and (B) total weight deposited as a function of deposited layers (1, 3, 5, 7 and 9 layers) for the films.

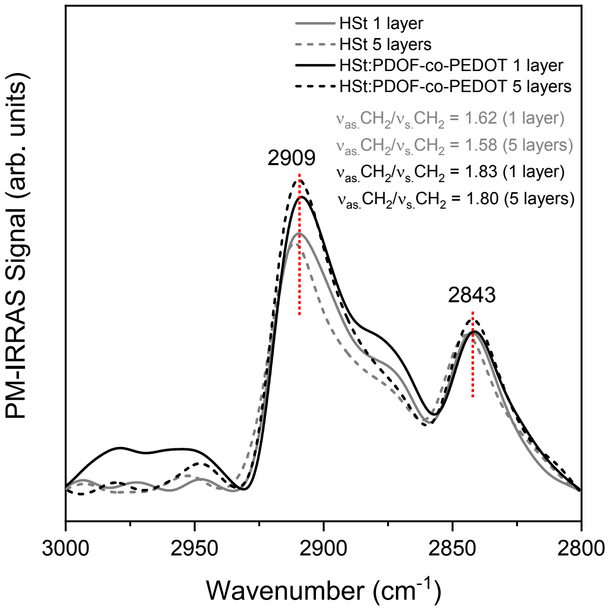

Figure S6. PM-IRRAS spectra of neat HSt and HSt:PDOF-co-PEDOT LB films, and respective order parameters.

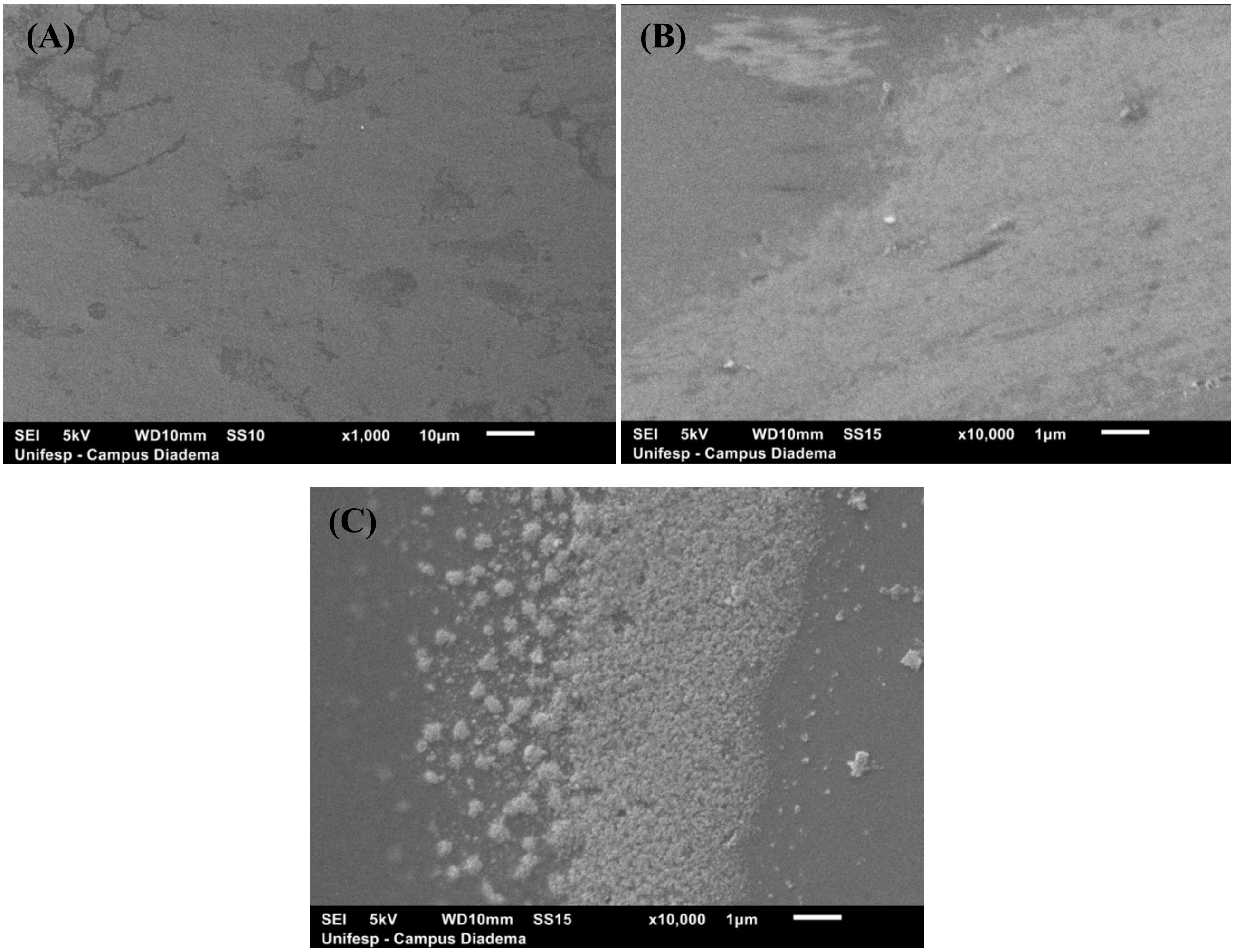

Figure S7. SEM images of 5-layers LB films of HSt:PDOF-co-PEDOT and HSt:PDOF-co-PEDOT/AgNP sph (A and B, respectively), compared to a 5-layers spin-coated film of PDOF-co-PEDOT/AgNP sph (C).

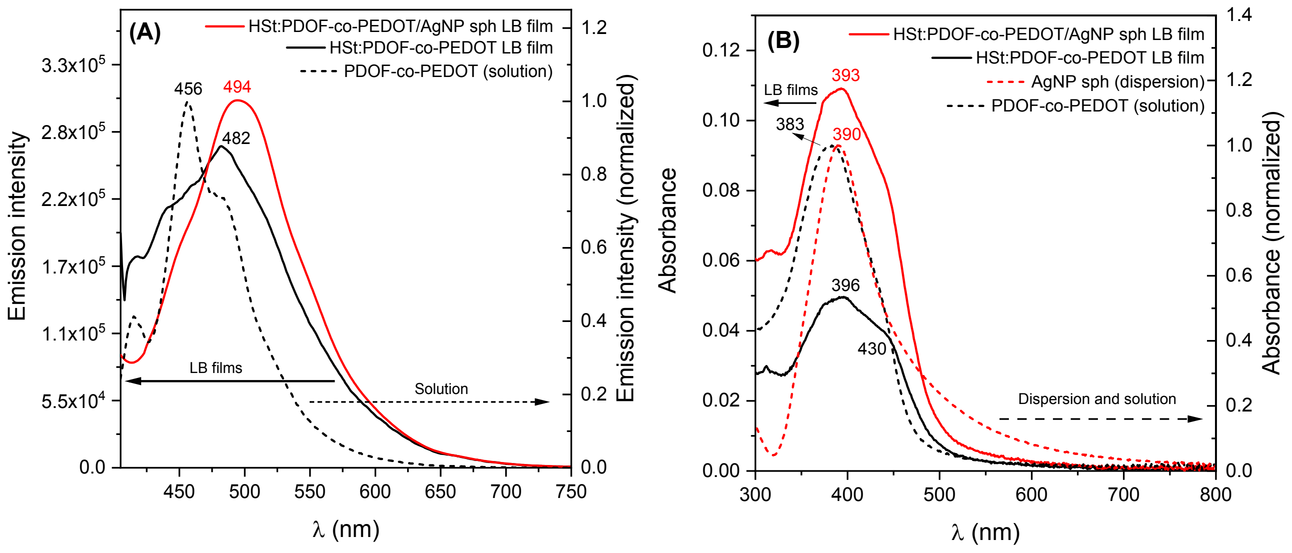

Figure S8. Emission (A) and absorption (B) spectra of LB films compared to normalized spectra (dashed curves) of PDOF-co-PEDOT (chloroform solution – 6 mg L^{-1}) and AgNP sph. (aqueous dispersion – 60 mg L^{-1}).
